# Association between hemodynamics, morphology, and rupture risk of intracranial aneurysms: a computational fluid modeling study

**DOI:** 10.1007/s10072-017-2904-y

**Published:** 2017-03-11

**Authors:** Tianlun Qiu, Guoliang Jin, Haiyan Xing, Haitao Lu

**Affiliations:** 1 Department of Neurosurgery, Shaoxing People’s Hospital, Shaoxing, 312000 Zhejiang China; 2 School of Medicine, Shaoxing University, Zhejiang, China; 3 Department of Neurosurgery, Chongming Branch of Shanghai Xinghua Hospital, Chongming, 202150 Shanghai China

**Keywords:** Intracranial aneurysms, Wall shear stress, Hemodynamics, Computational fluid dynamics

## Abstract

The objective of the study was to examine the correlations between intracranial aneurysm morphology and wall shear stress (WSS) to identify reliable predictors of rupture risk. Seventy-two intracranial aneurysms (41 ruptured and 31 unruptured) from 63 patients were studied retrospectively. All aneurysms were divided into two categories: narrow (aspect ratio ≥1.4) and wide-necked (aspect ratio <1.4 or neck width ≥4 mm). Computational fluid dynamics was used to determine the distribution of WSS, which was analyzed between different morphological groups and between ruptured and unruptured aneurysms. Sections of the walls of clipped aneurysms were stained with hematoxylin–eosin, observed under a microscope, and photographed. Ruptured aneurysms were statistically more likely to have a greater low WSS area ratio (LSAR) (*P* = 0.001) and higher aneurysms parent WSS ratio (*P* = 0.026) than unruptured aneurysms. Narrow-necked aneurysms were statistically more likely to have a larger LSAR (*P* < 0.001) and lower values of MWSS (*P* < 0.001), mean aneurysm-parent WSS ratio (*P* < 0.001), HWSS (*P* = 0.012), and the highest aneurysm-parent WSS ratio (*P* < 0.001) than wide-necked aneurysms. The aneurysm wall showed two different pathological changes associated with high or low WSS in wide-necked aneurysms. Aneurysm morphology could affect the distribution and magnitude of WSS on the basis of differences in blood flow. Both high and low WSS could contribute to focal wall damage and rupture through different mechanisms associated with each morphological type.

**Electronic supplementary material:**

The online version of this article (doi:10.1007/s10072-017-2904-y) contains supplementary material, which is available to authorized users.

## Introduction

Aneurysm rupture is the most common cause of non-traumatic subarachnoid hemorrhage. Hemodynamics are recognized as some of the many factors responsible for aneurysm rupture [1]. Computational fluid dynamics (CFD) is a branch of fluid mechanics that uses numerical analysis and data structures to solve and analyze problems that involve fluid flows. Image-based computational fluid dynamics (CFD) modeling identified associations between the hemodynamics of intracranial aneurysms and the likelihood of their growth and rupture [2], highlighting the promising possibility that aneurysmal hemodynamics may provide objective metrics to improve the stratification of rupture risk [3], but the growing number of proposed hemodynamic parameters makes it difficult to establish a consensus on this issue [4–6].

Wall shear stress (WSS) means the tangential drag force produced by horizontal movement of the fluid across the surface. In blood vessel, WSS acts on endothelium and is the mechanical force responsible for the acute change in luminal diameter. Currently, conflicting results concerning aneurysmal WSS, the most frequently explored hemodynamic parameter of augmented rupture risk, puzzle the IA research and clinical communities. Both high and low aneurysmal WSS are correlated with intracranial aneurysm growth and rupture [5], but disparate findings typically fall into four categories: (1) high spatial mean WSS (MWSS) is associated with rupture [7]; (2) low spatial MWSS is associated with rupture [2, 8, 9]; (3) high maximum WSS is associated with rupture [10, 11]; and (4) WSS is not associated with rupture [12–14].

Valencia et al. [15] observed an association between the mean WSS at the aneurysmal sac and aneurysm surface index in lateral unruptured and ruptured aneurysms. 4D-flow magnetic resonance imaging was used to demonstrate that large/giant saccular aneurysms and small saccular aneurysms have higher peak velocities and WSS compared with fusiform aneurysms [16]. WSS was increased in giant saccular aneurysms, indicating a relationship between aneurysm size and hemodynamics. There is an increase in aneurysm area exposed to low WSS once the aneurysm size ratio (ASR) is decreased and the flow became slower [17].

With the above discrepancies and lack of consensus in mind, the objective of this study was to analyze whether WSS was significantly different in ruptured and unruptured aneurysms, whether WSS was significantly different in aneurysms with two disparate aspect ratios, and whether the hemodynamics are significantly different between the two groups.

## Materials and methods

### Study design and patients

This was a retrospective study of the imaging data of 72 aneurysms in 63 patients from the hospital taken from January 2012 to December 2015. Forty-one patients were hospitalized for acute headache and diagnosed with subarachnoid hemorrhage (SAH) by CT scan. Twenty-two patients with unruptured aneurysms were admitted for one or more of the following reasons: (1) aneurysms diagnosed on clinical investigation of headaches, transient ischemic attack, stroke, or seizures; (2) incidentally found on imaging; (3) incidentally found during hospitalization for another condition (e.g., head trauma); (4) signs and symptoms of cranial compression; or (5) sentinel headache (without evidence of SAH on CT or lumber puncture).

The patient exclusion criteria were: (1) malignant tumor; (2) malignant hypertension; or (3) any serious systemic disease. The aneurysm exclusion criteria were: (1) false aneurysm; (2) inflammatory aneurysm; (3) traumatic aneurysm; (4) dissecting aneurysm; or (5) aneurysm with arteriovenous malformation. The multiple aneurysms with unascertainable responsible aneurysms were also excluded.

The study protocol was approved by the ethics committee of the hospital. Informed consent was obtained from all patients. (Ethical approval number: 2016-28).

### Imaging

All catheter angiograms were performed using standard transfemoral catheterization of the cerebral vessels, and digital subtraction angiography was performed using a Philips Integris Biplanar Unit (Philips Medical Systems, Best, The Netherlands). Rotational angiograms were obtained using a 6-s constant injection of contrast agent and a 180° rotation with imaging at 15 frames/s for a total of 8 s. Data from these images were transferred to the Philips Integris Workstation and reconstructed into 3D voxel data using the standard proprietary software provided with the system. 3D digital reconstruction that did not contain the entire aneurysm or associated parent arteries was excluded. The location of each aneurysm was recorded. Measurements of the aneurysm dome, neck, and the associated parent artery were performed using the conventional angiographic images and reference markers included in the view.

### Morphological assessments of aneurysms

Two-dimensional (2D) variables were measured by digital subtraction angiography: height (the maximum distance from the center of the aneurysm neck and the dome of the aneurysm) and neck width (parallel to the parent artery). All aneurysms were divided into two categories: narrow-necked (aspect ratio ≥1.4) and wide-necked (aspect ratio <1.4 or neck width ≥4 mm).

### Hemodynamic models

Meshes were imported into the simulation software Fluent15 (ANSYS Inc., Canonsburg, PA, USA). The measured inlet flow rate was applied at the cervical segment of the internal carotid artery and far away from the aneurysm so that the flow within the aneurysm was not sensitive to the inflow boundary condition [18]. Blood was modeled as an incompressible Newtonian fluid with a density of *ρ* = 1050 kg/m^3^ and a viscosity of 3.5 × 10^−3^ N/m^2^ s. The governing equations were the unsteady state Navier–Stokes equation and the continuity equation in 3D. The given boundary conditions were the no-slip and rigid condition on the wall, flat velocity profile at the inlet, and zero pressure at the outlet. Patient-specific flow conditions were applied for the inlet, which were obtained postoperatively from transcranial Doppler measurement (mean flow rate of 185 ml/min; maximum flow rate of 301 ml/min; heart rate of 64 bpm; mean Reynolds number of 364; Womersley number of 2.32). The governing equations were solved using a finite volume method and by applying the SIMPLE method for pressure–velocity coupling [19, 20]. Three pulsatile cycles were simulated to ensure that numeric stability had been reached, and the last cycle was taken as output.

### Hemodynamic parameter calculation

WSS was calculated from the simulated flow fields of each aneurysm. The WSS for pulsatile flow was calculated by integrating the WSS magnitude at each node over the cardiac cycle (Eq. 1):

1 $${\text{WSS}} = \frac{1}{T}\int\limits_{0}^{T} {\left| {{\text{wss}}_{i} } \right|} {\text{d}}t$$

The highest WSS (HWSS) was defined as the highest magnitude of intra-aneurysmal WSS. Mean WSS (MWSS) was the average of the WSS over the entire aneurysmal surface. Low WSS area ratio (LSAR) was defined as the area of the aneurysm wall exposed to a WSS below 10% of the mean parent arterial WSS normalized by the dome area [12].

Mean aneurysm-parent vessel WSS ratio (M-P WSS ratio): MWSS/PWSS.

Highest aneurysm-parent vessel WSS ratio (H-P WSS ratio): HWSS/PWSS.

PWSS (mean WSS of the near parent vessel) (1.0 cm away from the aneurysm neck, Supplementary Fig. 1) in a cardiac cycle was measured in all 72 events. The MWSS and HWSS of the aneurysm and PWSS and the low WSS area (LSA) of the aneurysm were measured in all 72 events.

### Pathology

Six wide-necked aneurysms (aspect ratio <1.4 or neck width ≥4 mm) were resected, fixed, and paraffin-embedded after clipping. All narrow-necked aneurysms (aspect ratio ≥1.4) were embolized using a Guglielmi detachable coil. The resected aneurysm wall was stained with hematoxylin–eosin and immunohistochemistry.

### Grouping

Aneurysms were divided into two groups: ruptured and unruptured. Because of the close correlation between aspect ratio and hemodynamic factors, all aneurysms were divided into those with a narrow neck (aspect ratio ≥1.4) and those with a wide neck (aspect ratio <1.4 or neck width ≥4 mm). To eliminate the influence of hemodynamic factors due to different aspect ratios and to compare the true hemodynamic factors, the aneurysms were divided into four groups: (1) ruptured and narrow-neck; (2) unruptured and narrow-necked; (3) ruptured and wide-necked; and (4) unruptured and wide-necked.

### Statistical analysis

Statistical analysis was performed using SPSS 16.0 (IBM, Armonk, NY, USA). Correlation analyses were performed to explore how the geometrical morphology of the aneurysms affected hemodynamics. The Smirnov–Kolmogorov test was performed to determine the normality of the distribution of the continuous variables. Normally distributed continuous variables were expressed as means ± standard deviations and analyzed using the independent-samples *t* test. Non-normally distributed variables were presented as medians and quartiles and analyzed using the Mann–Whitney *U* test. Two-sided *P* values <0.05 were considered statistically significant.

Independent-sample *t* tests and Mann–Whitney *U* tests were performed between groups to confirm which hemodynamic factor was the most important index of aneurysm rupture. Binary logistic regression was performed separately using regression to assess the hemodynamic features that achieved univariate statistical significance (*P* < 0.05). Spearman correlation coefficients were performed between hemodynamic and geometrical morphology factors by bivariate correlation analysis.

## Results

### Characteristics of the patients

There were 21 men and 42 women. The patients were 34–82 years of age (mean 56.9 ± 9.43 years).

### Characteristics of the aneurysms

Three patients had two aneurysms, and three patients had three aneurysms. Of the selected aneurysms, 18 were located in the medial cerebral artery, 21 in the carotid artery, 26 in the posterior communicating artery, three in the anterior cerebral artery, and four in the ophthalmic artery. Anterior communicating artery aneurysms (which are visualized by two inflow injections with different directions) and vertebral basilary artery aneurysms with different diameter and blood flow distribution were excluded. Forty-one aneurysms were ruptured and 31 were unruptured. Aneurysm height ranged from 2.5 to 15.7 mm, while the height-to-neck ratio ranged from 0.49 to 3.10.

### Correlations according to aneurysm rupture

Ruptured aneurysms were more likely to have a greater LSAR (*P* = 0.001) and higher aneurysms parent WSS ratio (*P* = 0.026) than unruptured aneurysms (Table 1). LSAR (*P* = 0.006) and the highest aneurysms parent WSS ratio (*P* = 0.023) were the hemodynamic factors predictive of rupture (Supplementary Table 1). MWSS (*r* = −0.558, *P* < 0.001), mean aneurysm-artery WSS ratio (*r* = −0.650, *P* < 0.001), HWSS (*r* = −0.267, *P* = 0.023), and the highest aneurysm-parent WSS ratio (*r* = −0.342, *P* = 0.003) were negatively correlated with aspect ratio. LSAR (*r* = 0.583, P < 0.001) was positively correlated with aspect ratio (Supplementary Fig. 2).

**Table 1:** Wall shear stress and its ratios for ruptured and unruptured aneurysms

Parameters	Ruptured	Unruptured	*P*
Mean aneurysm WSS (Pa)			
Median	3.15	4.09	0.071
Quartiles	2.80	2.05	
Mean parent artery WSS (Pa)			
Median	6.83	6.55	0.909
Quartiles	6.27	4.37	
Low shear area ratio (low shear area/dome area)			
Median	0.09	0.020	0.001
Quartiles	0.18	0.050	
Highest aneurysm WSS (Pa)			
Median	10.07	8.66	0.384
Quartiles	8.89	4.12	
Mean aneurysm-parent WSS ratio	0.511 ± 0.293	0.606 ± 0.186	0.101
Highest aneurysm-parent WSS ratio	1.439 ± 0.421	1.258 ± 0.252	0.026

Normally distributed variables are presented as mean ± SD and were analyzed with the independent-samples *t* test. Non-normally distributed variables are presented as medians and quartiles and were analyzed using the Mann–Whitney *U* test. A *P* value <0.05 was deemed statistically significant

*WSS* wall shear stress

### Correlations according to aspect ratio

Narrow-necked (aspect ratio ≥1.4) aneurysms were more likely to have a larger LSAR (*P* < 0.001) and lower values of MWSS (*P* < 0.001), mean aneurysm-parent WSS ratio (*P* < 0.001), HWSS (*P* = 0.012), and the highest aneurysm-parent WSS ratio (*P* < 0.001) than wide-necked aneurysms (aspect ratio <1.4 or neck width ≥4 mm) (Table 2).

**Table 2:** Wall shear stress for narrow and wide-necked aneurysms

Parameters	Narrow	Wide	*P*
Mean aneurysm WSS (Pa)			
Median	2.37	4.33	<0.001
Quartiles	2.20	3.69	
Mean parent artery WSS (Pa)			
Median	6.28	6.83	0.484
Quartiles	5.69	4.31	
Low shear area ratio (low shear area/dome area)			
Median	0.13	0.020	<0.001
Quartiles	0.30	0.078	
Highest aneurysm WSS (Pa)			
Median	7.58	9.23	0.012
Quartiles	6.36	5.34	
Mean aneurysm-parent WSS ratio	0.327 ± 0.181	0.658 ± 0.214	<0.001
Highest aneurysm-parent WSS ratio	1.128 ± 0.379	1.470 ± 0.309	<0.001

Normally distributed variables are presented as mean ± SD and were analyzed with the independent-samples *t* test. Non-normally distributed variables are presented as medians and quartiles and were analyzed using the Mann–Whitney *U* test. A *P* value <0.05 was deemed statistically significant

*WSS* wall shear stress

### Correlations according to morphological categories

**Fig. 1:**
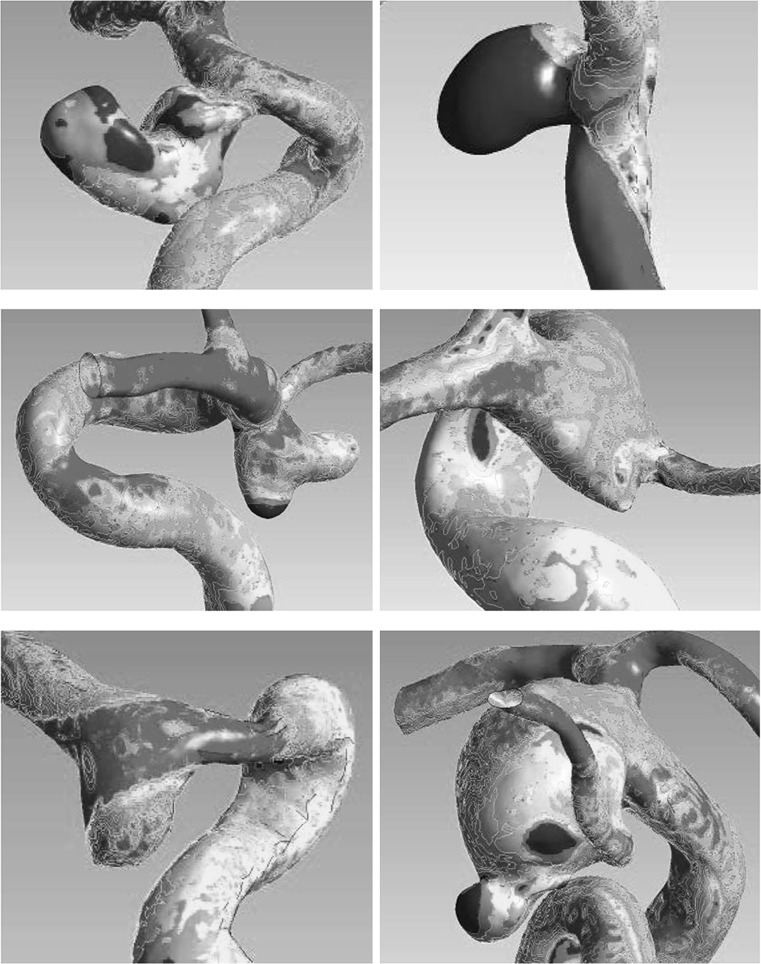
*Top* ruptured and narrow-neck aneurysm with a high ratio of low wall shear stress area. *Middle and bottom* ruptured and wide-neck aneurysm with a high ratio of low or highest wall shear stress area

**Fig. 2:**
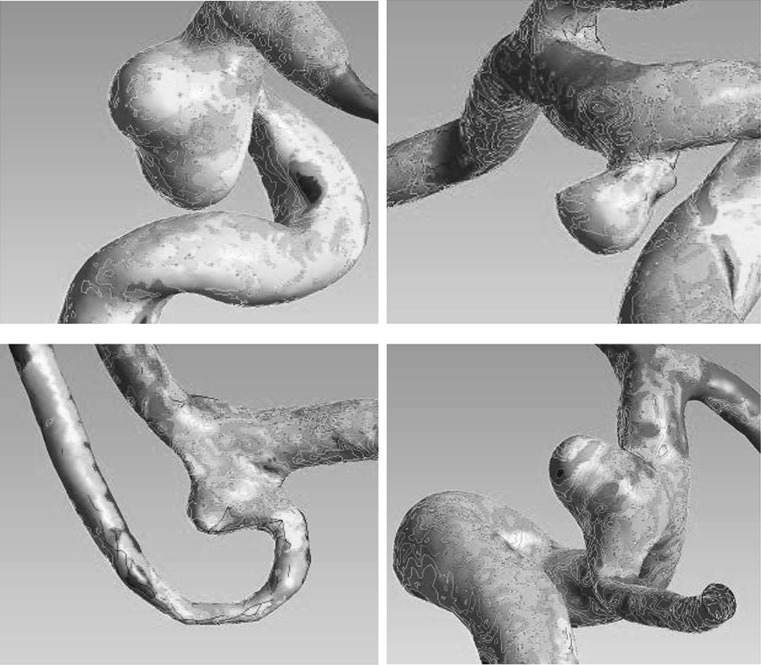
*Top* unruptured and narrow-neck aneurysm with a low ratio of low wall shear stress area. *Bottom* unruptured and wide-neck aneurysm with a low value of highest wall shear stress and no ratio of low wall shear stress area

In narrow-necked aneurysms, ruptured aneurysms were more likely to have larger LSAR (*P* = 0.015) and lower values of MWSS (*P* = 0.028) and mean aneurysm-parent WSS ratio (*P* = 0.001) than unruptured aneurysms (Table 3). Backward stepwise binary logistic regression indicated that mean aneurysm-parent WSS ratio (*P* = 0.018) was the only hemodynamic factor significantly predictive of rupture (Supplementary Table 2). In wide-necked aneurysms, ruptured aneurysms were more likely to have larger LSAR (*P* = 0.007), HWSS (*P* = 0.048), and the highest aneurysm-parent WSS ratio (*P* < 0.001) than unruptured aneurysms (Table 4). LSAR and the highest aneurysm-parent WSS ratio (both *P* = 0.003) were the only hemodynamic factors predictive of rupture (Supplementary Table 3). On average, ruptured aneurysms seemed to have a greater area of lower WSS compared with the parent artery in narrow-necked aneurysms (Fig. 1), while ruptured aneurysms seemed to have a greater degree of WSS diversity and area of the higher WSS or lower WSS compared with the parent artery in wide-necked aneurysms (Fig. 1). The WSS of the surface area in unruptured aneurysms was not extreme (Fig. 2).

**Table 3:** Wall shear stress for ruptured and unruptured aneurysms in the narrow-necked aneurysm group

Parameters	Ruptured	Unruptured	*P*
Mean aneurysms WSS (Pa)	1.96 ± 1.30	3.33 ± 1.45	0.028
Highest aneurysm WSS (Pa)	9.08 ± 6.64	8.19 ± 3.69	0.720
Mean parent artery WSS (Pa)	7.85 ± 3.98	7.14 ± 3.08	0.655
Low shear area ratio (low shear area/dome area)	0.340 ± 0.308	0.093 ± 0.125	0.015
Highest aneurysm-parent WSS ratio	1.098 ± 0.388	1.174 ± 0.382	0.649
Mean aneurysm-parent WSS ratio	0.233 ± 0.120	0.474 ± 0.164	0.001

Normally distributed variables are presented as mean ± SD and were analyzed with the independent-samples *t* test. A *P* value <0.05 was deemed statistically significant

*WSS* wall shear stress

**Table 4:** Wall shear stress for ruptured and unruptured aneurysms in wide-necked aneurysms

Parameters	Ruptured	Unruptured	*P*
Mean aneurysm WSS (Pa)			
Median	4.04	4.44	0.587
Quartiles	5.24	2.96	
Highest aneurysm WSS (Pa)	14.58 ± 10.93	9.95 ± 3.73	0.048
Mean parent artery WSS (Pa)	8.94 ± 6.13	7.73 ± 2.53	0.355
Low shear area ratio (low shear area/dome area)	0.075 ± 0.082	0.026 ± 0.032	0.007
Highest aneurysm-parent WSS ratio	1.616 ± 0.320	1.292 ± 0.176	0.000
Mean aneurysm-parent WSS ratio	0.656 ± 0.247	0.660 ± 0.170	0.954

Normally distributed variables are presented as mean ± SD and were analyzed with the independent-samples *t* test. Non-normally distributed variables are presented as medians and quartiles and were analyzed using the Mann–Whitney *U* test. A *P* value <0.05 was deemed statistically significant

*WSS* wall shear stress

### Pathological examination

The aneurysm wall showed two different types of pathological change after hematoxylin and eosin staining. The first type was characterized by a high number of degenerate smooth muscle cells (SMCs) producing collagen, with only sparse vascular endothelial cells and incomplete internal elastic lamina. No inflammatory response or atherosclerotic plaques were present in the high WSS aneurysms (Supplementary Fig. 3).

The second type was characterized by a large number of neutrophil granulocytes infiltrating the vessel wall, SMCs with vacuoles and mucoid degeneration, hyperplasia of collagenous fibers in the matrix, internal elastic lamina, scarce vascular endothelial cells, and a large volume of thrombotic material in the low WSS aneurysms wall (Supplementary Fig. 4). Few vascular endothelial cells were observed after immunohistochemistry for CD31 in the high WSS aneurysms (Supplementary Fig. 5), but a large number of neutrophil granulocytes infiltrating the vessel wall were observed after immunohistochemistry for CD68 in the low WSS aneurysms (Supplementary Fig. 6).

## Discussion

Meng et al. [21] suggested that the “high-versus-low WSS” controversy is a manifestation of the complexity of aneurysm pathophysiology. Low WSS and high oscillatory shear index trigger an inflammatory cell-mediated pathway that could be associated with the growth and rupture of large, atherosclerotic aneurysms, while high WSS combined with a positive WSS gradient triggers a mural cell-mediated pathway that could be associated with the growth and rupture of small or secondary bleb aneurysm [21]. Yu et al. [22] showed that ruptured aneurysms showed higher mean WSS and maximum WSS than unruptured aneurysms. It is assumed that a WSS of >2.0 N/m^2^ is suitable for maintaining the structure of the arterial vessels and that a WSS lower than 1.5 N/m^2^ will lead to the degeneration of endothelial cells via apoptosis [23]. In the present study, the WSS in the LSA was obviously lower than 1.5 N/m^2^. The highest aneurysm-parent WSS ratio was also a hemodynamic factors leading to aneurysm rupture, which is consistent with the study by Meng et al. [21].

In clinical practice, aneurysmal geometry has been extensively used to estimate the likelihood of intracranial aneurysm rupture [24–27]. Amenta et al. [24] showed that an AR of 1.6, a dome diameter of 10 mm, a deviated neck, and right-sidedness are independently associated with aneurysm rupture [24]. A study of multiple aneurysms in a single patient showed that aneurysms >4 mm had a higher maximum WSS and area of low WSS, while aneurysms <4 mm had a lower maximum WSS and area of low WSS [28]. There are many contradictory results in the literature. Aneurysm morphology itself has not been shown to lead to rupture. In the present study, hemodynamic variables were related to the geometric morphology of the aneurysm. A recent study showed that high flow impingement and WSS were associated with recanalization and regrowth, while low WSS was associated with aneurysm rupture [29].

Aspect ratio is negatively correlated with MWSS, mean aneurysm-artery WSS ratio, HWSS, and the highest aneurysm-parent WSS ratio, and positively correlated with LSAR. We found that aneurysms with an aspect ratio <1.4 or a neck width ≥4 mm (wide neck) had more inflow jet at the dome and a larger flow impingement region than aneurysms with an aspect ratio ≥1.4. Aneurysms with an aspect ratio <1.4 or a neck width ≥4 mm had significantly higher MWSS, mean aneurysm-artery WSS ratio, HWSS, the highest aneurysm-parent WSS ratio, and less LSAR than aneurysms with an aneurysm with aspect ratio ≥1.4.

The results of the present study strongly suggest that both high and low WSS were able to cause rupture in wide-necked aneurysms. Occasionally distinct high and low WSS could be found in the same aneurysm. The highest aneurysm-parent WSS ratio and LSAR were the only differentiating factors in wide-necked aneurysms according to the binary logistic regression analyses. We assume that different parts of the intracranial vessel are unequally resistant to high WSS. Highest aneurysm-parent WSS ratio seemed to be a more sensitive factor than simple HWSS. Unruptured aneurysms with either wide or narrow necks had similar characteristics in that no distinct observations of high and low WSS were made.

According to Meng et al. [3], aneurysm lesion presentation is highly heterogeneous in almost every observable metric. The first type (type I) involves high WSS and a positive WSS gradient (spatial derivative of WSS along the flow direction with respect to the streamwise distance) [21, 30]. Through endothelial cell mechanotransduction, these hemodynamic stresses initiate biochemical cascades when they exceed certain thresholds, leading to the local production and activation of proteases (the most important of which is matrix metalloproteinase) by wall cells [31], massive internal elastic lamina damage [30], and apoptosis [31], which are collectively responsible for media thinning and bulge formation [21]. Interestingly, inflammatory cell infiltration has not been observed in early stage intracranial aneurysm initiation [31].

In the second type (type II), the flow environment is likely to be dominated by low and oscillating WSS. This condition is exacerbated if secondary vortices form and/or flow instability increases [32]. Endothelial cells produce reactive oxygen species, up-regulate surface adhesion molecules and cytokines in the vessel wall, and increase luminal permeability [33, 34]. These inflammatory infiltrates can produce large amounts of matrix metalloproteinases that degrade the extracellular matrix [35], thus tipping the balance between eutrophic and degradative processes and driving intracranial aneurysm growth and rupture [36]. Furthermore, such “disturbed flow” environments promote the formation of atherosclerotic plaques [36, 37]. The formation of a luminal thrombus can further trap macrophages and neutrophils and harbor proteases, reactive oxygen species, and oxidized low-density lipoproteins [38]. These two pathological types depend on the WSS distribution of the aneurysm. These differences indicate the close relationship between hemodynamic status and pathological change.

## Limitations

Analysis of aneurysm shape to identify rupture factors relies on the hypothesis that aneurysm shape does not change after rupture. Even though increasing evidence indicates that such change does not occur [39], conclusive data on this subject remain scant. The pathological description of the aneurysms was made on only six samples. The vascular basement membrane and any extracellular matrix metalloproteinase were not visualized. There was the potential for some selection bias in the histological findings. Our CFD approach assumed rigid walls and did not take into account the viscoelasticity of the vessel wall because of the limited availability of physical information regarding arterial wall properties, such as elasticity and thickness. Finally, the affected artery was not taken into account in the analyses and a recent study suggested that the parameters affecting aneurysm rupture may depend on the artery harboring the aneurysm [40].

## Conclusion

Aneurysm morphology could affect the distribution and magnitude of WSS on the basis of differences in blood flow. Both high and low WSS could contribute to focal wall damage and rupture through different mechanisms associated with each morphological type.

## Electronic supplementary material

Below is the link to the electronic supplementary material.

Supplementary material 1 (TIFF 326 kb) **Supplementary Fig.** **1.** Diagram of the aneurysm measurement method. The height of the aneurysm was defined as the longest distance from the center of the neck to the dome tip. Neck width was measured as the width parallel to the parent artery. In this diagram, the near vessel is 1.0 cm away from the aneurysm neck

Supplementary material 2 (TIFF 357 kb) **Supplementary Fig.** **2.** Spearman correlations were performed between hemodynamic and geometrical morphology factors. Aspect ratio is positively correlated to low wall shear stress (WSS) area ratio (LSAR) (r = 0.583) and negatively correlated to the highest WSS (r = -0.267), mean WSS (r = -0.558), mean aneurysm-artery WSS ratio (r = -0.650), and the highest aneurysm-parent WSS ratio (r = -0.342). LSAR and mean aneurysm-artery WSS ratio had the strongest correlations to aspect ratio

Supplementary material 3 (TIFF 930 kb) **Supplementary Fig.** **3.** A large number of degenerate smooth muscle cells producing collagen and only sparse vascular endothelial cells and incomplete internal elastic lamina can be observed. No inflammatory response or atherosclerotic plaque were observed in this series of aneurysms

Supplementary material 4 (TIFF 1733 kb) **Supplementary Fig.** **4.** Hematoxylin & eosin staining showing a large number of neutrophil granulocytes infiltrating the vessel wall. Smooth muscle cells with vacuoles and mucoid degeneration, collagenous fibers hyperplasia  in the matrix, and internal elastic lamina can be seen. Vascular endothelial cells are scarce. A large amount of thrombotic material is present in the aneurysm wall

Supplementary material 5 (TIFF 538 kb) **Supplementary Fig.** **5.** Sparse vascular endothelial cells visualized by immunohistochemistry for CD31

Supplementary material 6 (TIFF 616 kb) **Supplementary Fig.** **6.** A large number of neutrophil granulocytes infiltrating the vessel wall visualized by immunohistochemistry for CD68

Supplementary material 7 (DOC 36 kb)
