# Supplementary figures and images for: Association between hemodynamics, morphology, and rupture risk of intracranial aneurysms: a computational fluid modeling study
Source: Neurol Sci. 2017 Mar 11;38(6):1009–18. doi: 10.1007/s10072-017-2904-y (PMC5486504; doi:10.1007/s10072-017-2904-y)

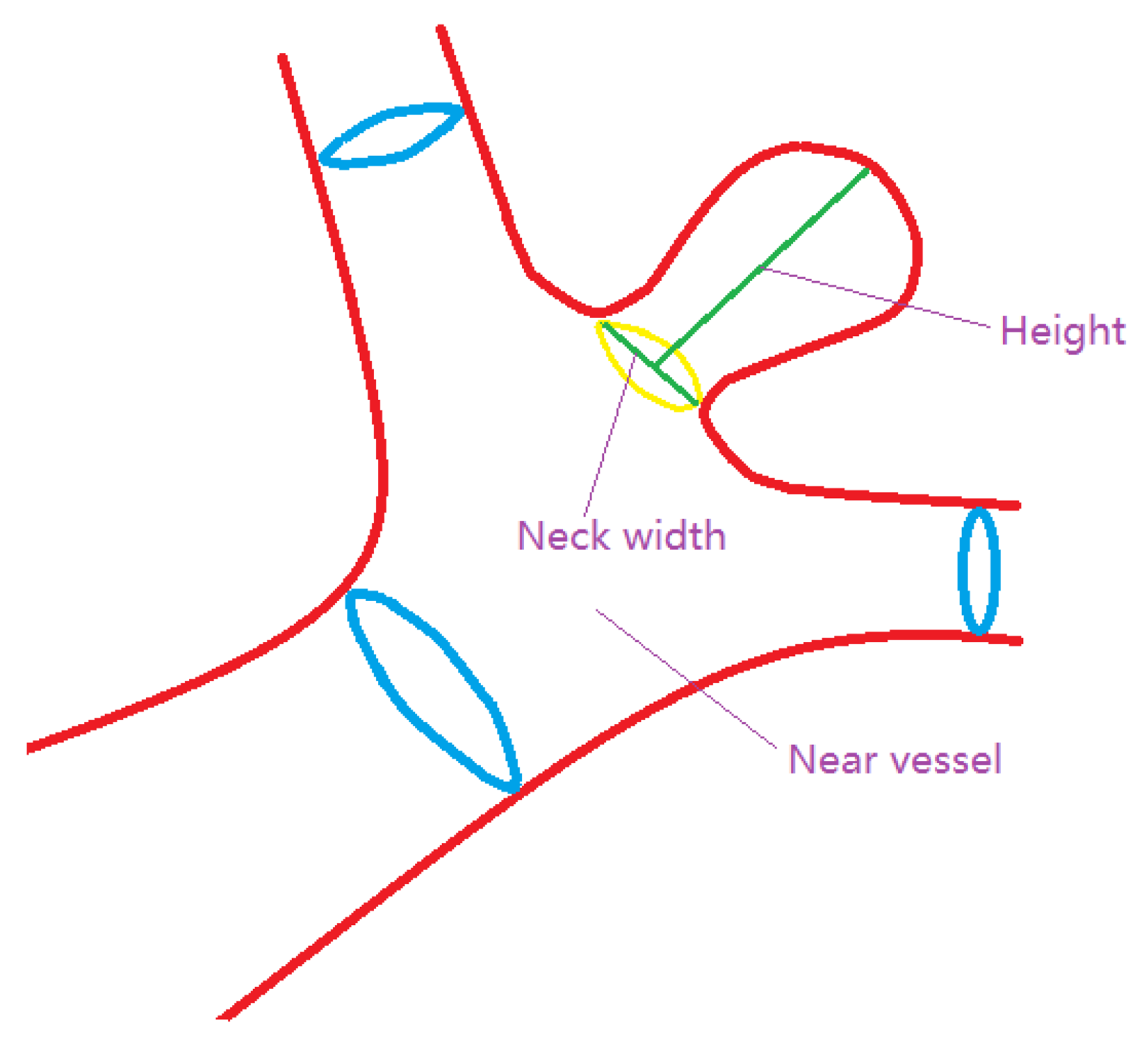

Supplement: Supplementary file 1 — Supplementary material 1 (TIFF 326 kb) Supplementary Fig. 1. Diagram of the aneurysm measurement method. The height of the aneurysm was defined as the longest distance from the center of the neck to the dome tip. Neck width was measured as the width parallel to the parent artery. In this diagram, the near vessel is 1.0 cm away from the aneurysm neck [file 10072_2017_2904_MOESM1_ESM.tif]

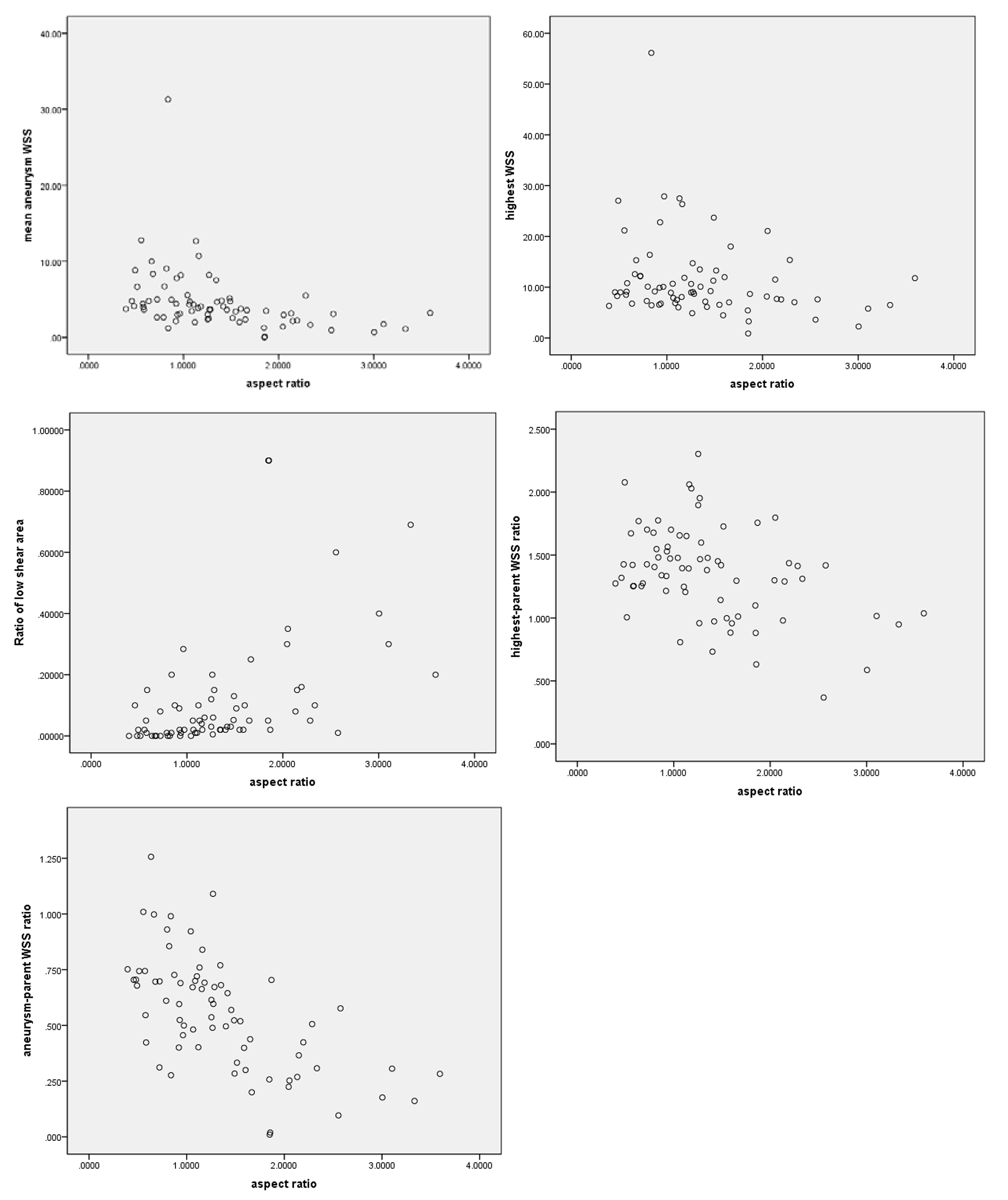

Supplement: Supplementary file 2 — Supplementary material 2 (TIFF 357 kb) Supplementary Fig. 2. Spearman correlations were performed between hemodynamic and geometrical morphology factors. Aspect ratio is positively correlated to low wall shear stress (WSS) area ratio (LSAR) (r = 0.583) and negatively correlated to the highest WSS (r = -0.267), mean WSS (r = -0.558), mean aneurysm-artery WSS ratio (r = -0.650), and the highest aneurysm-parent WSS ratio (r = -0.342). LSAR and mean aneurysm-artery WSS ratio had the strongest correlations to aspect ratio [file 10072_2017_2904_MOESM2_ESM.tif]

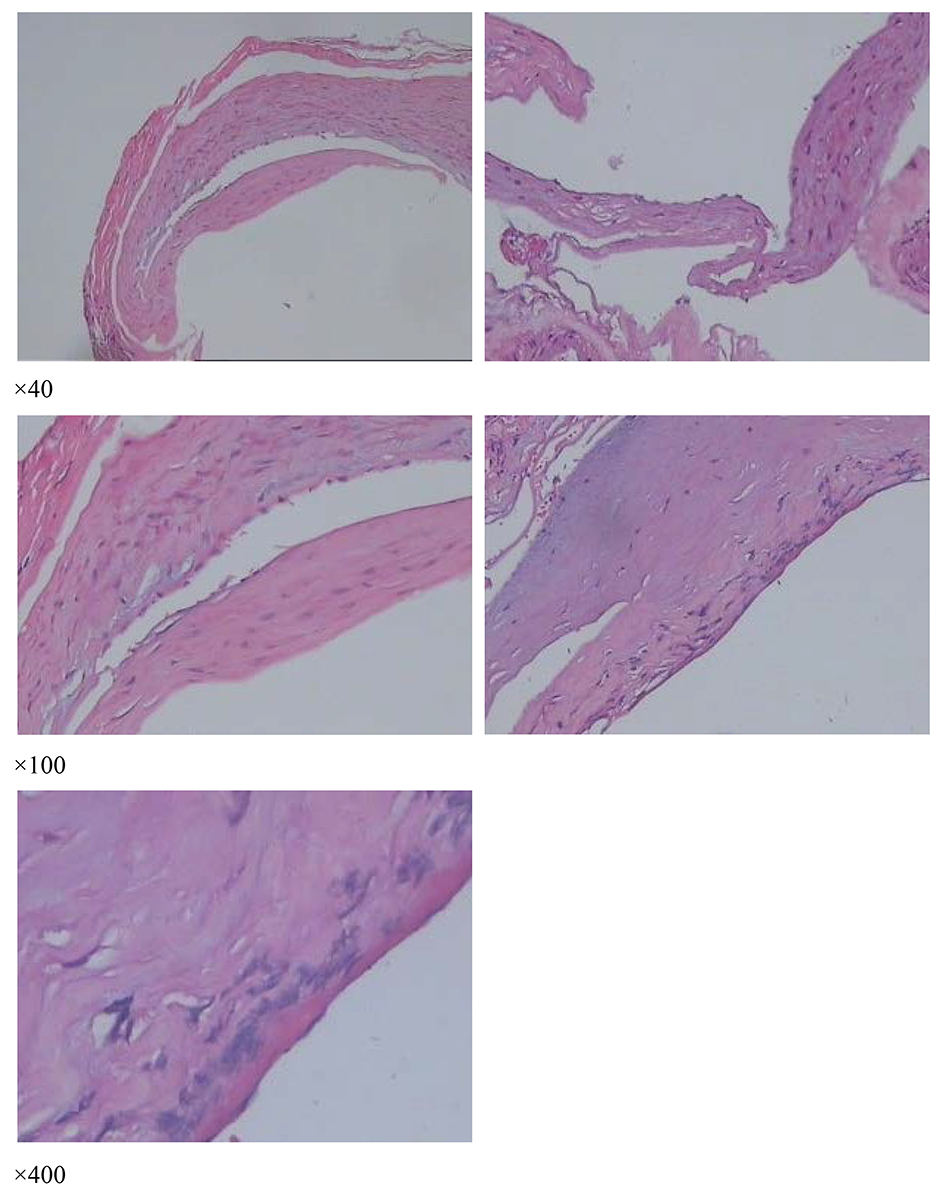

Supplement: Supplementary file 3 — Supplementary material 3 (TIFF 930 kb) Supplementary Fig. 3. A large number of degenerate smooth muscle cells producing collagen and only sparse vascular endothelial cells and incomplete internal elastic lamina can be observed. No inflammatory response or atherosclerotic plaque were observed in this series of aneurysms [file 10072_2017_2904_MOESM3_ESM.tif]

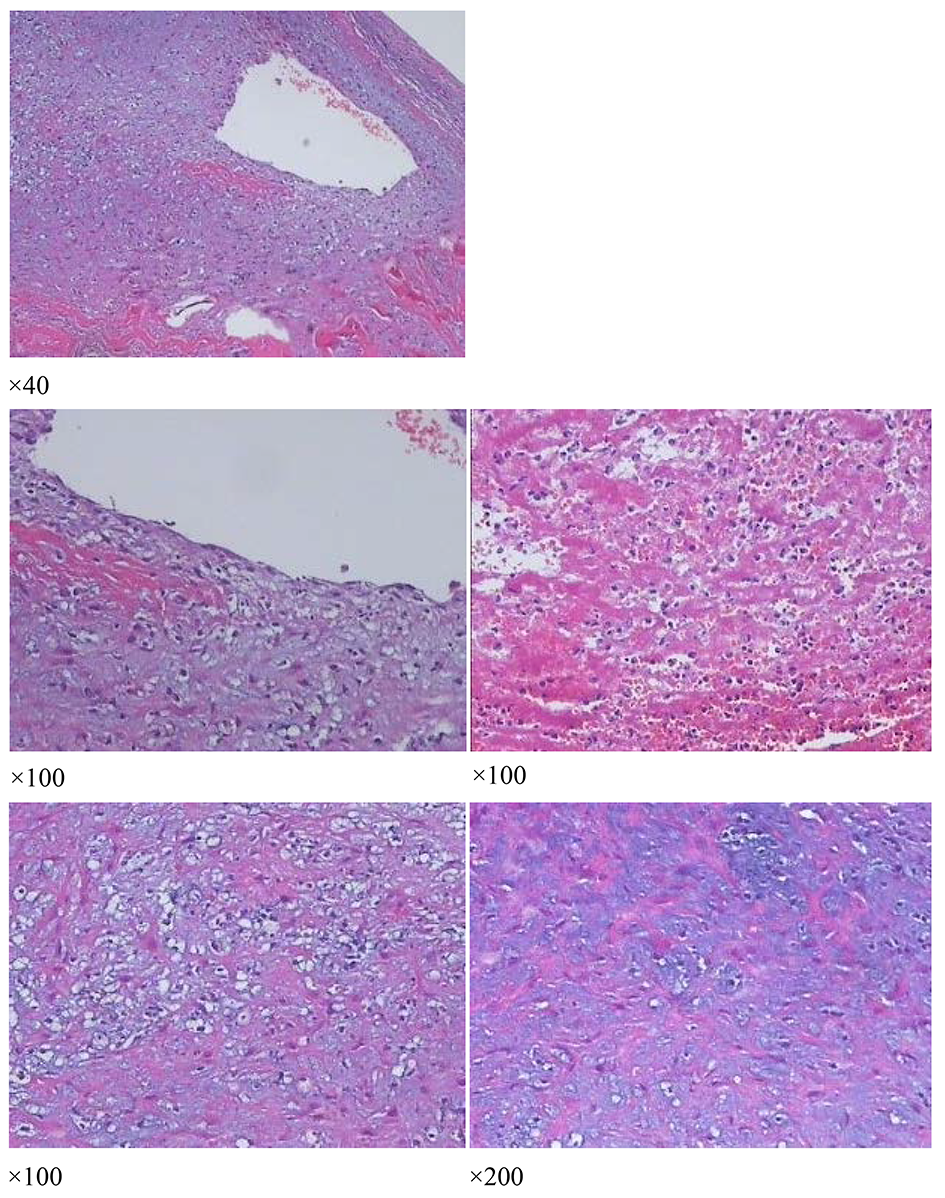

Supplement: Supplementary file 4 — Supplementary material 4 (TIFF 1733 kb) Supplementary Fig. 4. Hematoxylin & eosin staining showing a large number of neutrophil granulocytes infiltrating the vessel wall. Smooth muscle cells with vacuoles and mucoid degeneration, collagenous fibers hyperplasia in the matrix, and internal elastic lamina can be seen. Vascular endothelial cells are scarce. A large amount of thrombotic material is present in the aneurysm wall [file 10072_2017_2904_MOESM4_ESM.tif]

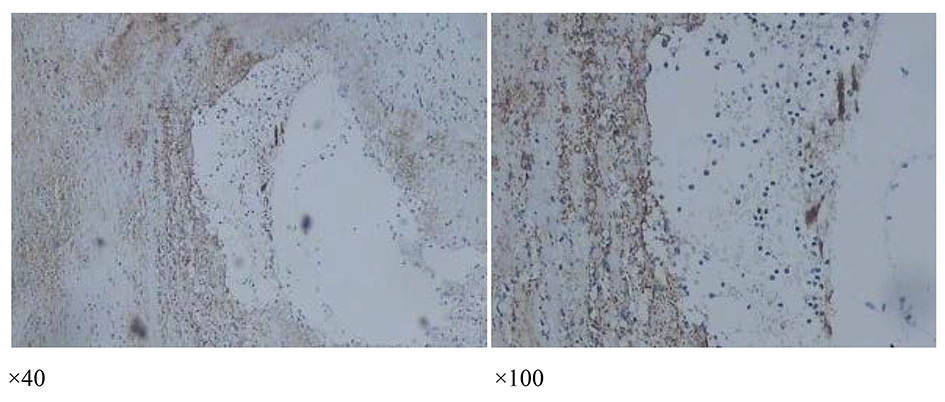

Supplement: Supplementary file 5 — Supplementary material 5 (TIFF 538 kb) Supplementary Fig. 5. Sparse vascular endothelial cells visualized by immunohistochemistry for CD31 [file 10072_2017_2904_MOESM5_ESM.tif]

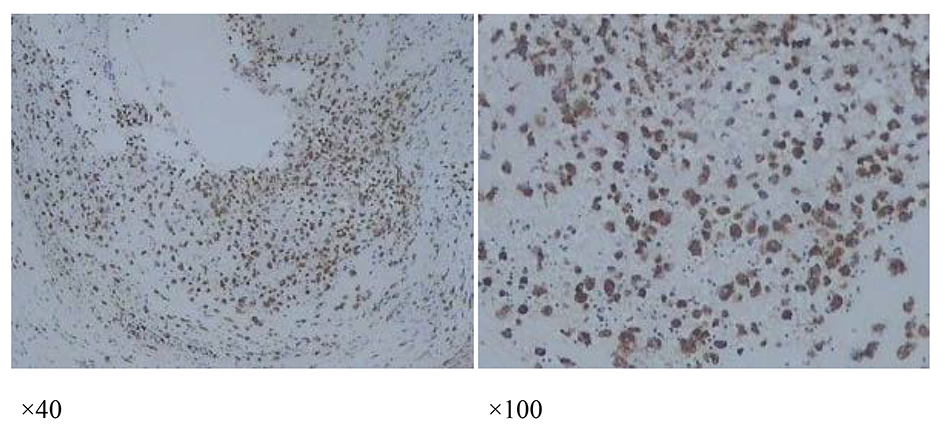

Supplement: Supplementary file 6 — Supplementary material 6 (TIFF 616 kb) Supplementary Fig. 6. A large number of neutrophil granulocytes infiltrating the vessel wall visualized by immunohistochemistry for CD68 [file 10072_2017_2904_MOESM6_ESM.tif]
