# Supplementary material for: Association between hemodynamics, morphology, and rupture risk of intracranial aneurysms: a computational fluid modeling study
Source: Neurol Sci. 2017 Mar 11;38(6):1009–18. doi: 10.1007/s10072-017-2904-y (PMC5486504; doi:10.1007/s10072-017-2904-y)
Supplement: Supplementary file 7 — Supplementary material 7 (DOC 36 kb) [file 10072_2017_2904_MOESM7_ESM.doc]

**Association between hemodynamics, morphology, and rupture risk of intracranial aneurysms: a computational fluid modeling study**

**Neurological Sciences**

Tianlun Qiu^1*^, Guoliang Jin^1^, Haiyan Xing^2^, Haitao Lu^3^

*^1^Department of Neurosurgery, Shaoxing People’s Hospital, Shaoxing 312000, Zhejiang, China*

*^2^School of Medicine, Shaoxing University, Zhejiang, China*

*^3^Department of Neurosurgery, Chongming Brach of Shanghai Xinghua Hospital, Chongming 202150, Shanghai, China*

**^*^Corresponding author:**

Tianlun Qiu

Department of Neurosurgery, Shaoxing People’s Hospital, Shaoxing 312000, Zhejiang, China

Tel: +86-13676884796

Fax: +86-21-64085875

E-mail: sxrqtl@163.com

**Supplementary Table 1.** Wall shear stress of ruptured and unruptured aneurysm by binary logistic regression analyses

| Parameters | B | | S.E | Walds | P | Odds ratio | 95%CI | |
| --- | --- | --- | --- | --- | --- | --- | --- | --- |
| Constant | 3.889 | | 1.598 | 5.923 | 0.015 | 48.872 |  |  |
| Low shear area ratio (%) (low shear area/dome area) | -9.887 | | 3.60 | 7.542 | 0.006 | 0.000 | 0.000 | 0.059 |
| Highest aneurysm-parent WSS ratio | | -1.763 | 0.776 | 5.167 | 0.023 | 0.172 | 0.038 | 0.784 |

WSS: wall shear stress; 95%CI: confidence interval.

**Supplementary Table 2.** Wall shear stress of ruptured and unruptured aneurysm in narrow-necked aneurysms by backward stepwise binary logistic regression analysis

| Parameters | B | S.E | Walds | P | Odds ratio | 95%CI | |
| --- | --- | --- | --- | --- | --- | --- | --- |
| Constant | -5.356 | 2.178 | 6.045 | 0.014 | 0.005 |  |  |
| Mean aneurysm-parent WSS ratio | 14.185 | 6.022 | 5.549 | 0.018 | 1.45*10^6^ | 10.83 | 1.93*10^11^ |

WSS: wall shear stress; 95%CI: confidence interval.

**Supplementary Table 3.** Wall shear stress of ruptured and unruptured aneurysm in wide-necked aneurysms by backward stepwise binary logistic regression analysis

| Parameters | B | S.E | Walds | P-value | Odds ratio | 95%CIs | |
| --- | --- | --- | --- | --- | --- | --- | --- |
| Constant | 28.636 | 9.678 | 8.754 | 0.003 | 2.731*10^12^ |  |  |
| Low shear area ratio (low shear area/dome area) | -58.909 | 19.563 | 9.068 | 0.003 | 0.000 | 0.000 | 0.000 |
| Highest aneurysm-parent WSS ratio | -18.150 | 6.212 | 8.537 | 0.003 | 0.003 | 0.000 | 0.003 |

WSS: wall shear stress; 95%CI: confidence interval.
